# Child Born Alive at the Fourth Month and a Half of Pregnancy

**Published:** 1847-04

**Authors:** 


*Child Born Alive at the Fourth Month and a Half of Pregnancy.*—M. Maisonneuve lately communicated to the Surgical Society the case of a female who aborted at the fourth month and a half of pregnancy. When he was called to her, the ovum had been expelled with its membranes for about two hours. On dividing the membranes and examining the fœtus, he found to his surprise that it was still moving. He applied warmth, and in some degree succeeded in resuscitating it, for in a few minutes the respiratory motions were performed with regularity, but the child died in about six hours.—*Jour. de Méd.*

